# Singular Instance of Depraved Appetite in a Monkey

**Published:** 1881-07

**Authors:** Frank J. Thompson

**Affiliations:** Zoological Garden, Cincinnati


### CORRESPONDENCE.

### SINGULAR INSTANCE OF DEPRAVED APPETITE IN A MONKEY.

EDITOR OF JOURNAL OF COMPARATIVE MEDICINE:

ON the morning of March 22d one of the bonnet monkeys (*Macaeus radiatus*) in the Society's collection was found prone on her stomach, in the bottom of the cage, in great pain. On making an examination, it was found that she was suffering from diarrhœa, and one-quarter grain of morphia was administered. By noon the pain seemed to be relieved, as she moved about the cage and took some food; but, as the discharges continued, a like dose of morphia was given. The next morning she ate as usual, nor could any signs of a discharge during the night be found. About noon she was siezed with violent convulsions, and died in about two hours. On dissecting her, the heart, lungs, and liver all appeared in a normal state, but the stomach and intestines were much swollen and inflamed. On opening the stomach, the enclosed mass of broom-straw tips was found, which, you will find, seem to have been swallowed without any attempt at chewing. She must have worked most industriously for a long while to pick up and swallow such a quantity of tips, which happened to break off the broom during the daily sweeping of the floor and dusting of the grating of the cage.

FRANK J. THOMPSON.

ZOOLOGICAL GARDEN, CINCINNATI.

[This curious specimen has been placed in the Museum of the Columbia Veterinary College.]

